# Who benefits from adjuvant chemotherapy? Identification of early recurrence in intrahepatic cholangiocarcinoma patients after curative-intent resection using machine learning algorithms

**DOI:** 10.3389/fonc.2025.1594200

**Published:** 2025-06-06

**Authors:** Qi Li, Hengchao Liu, Yubo Ma, Zhenqi Tang, Chen Chen, Dong Zhang, Zhimin Geng

**Affiliations:** Department of Hepatobiliary Surgery, The First Affiliated Hospital of Xi’an Jiaotong University, Xi’an, Shaanxi, China

**Keywords:** intrahepatic cholangiocarcinoma, recurrence, prognosis, machine learning, adjuvant chemotherapy

## Abstract

**Objective:**

It is vital to enhance the identification of early recurrence in intrahepatic cholangiocarcinoma (ICC) patients after curative-intent resection and to determine which patients could benefit from adjuvant chemotherapy (ACT). This study aimed to evaluate the effectiveness of machine learning algorithms in detecting early recurrence in ICC patients and select those who would benefit from ACT to improve prognosis.

**Methods:**

The study analyzed 254 intrahepatic cholangiocarcinoma (ICC) patients who underwent curative-intent resection to identify early recurrence predictors. Through logistic regression and feature importance analysis, we determined key risk factors and subsequently developed machine learning models utilizing the top five predictors for early recurrence prediction. The predictive performance was validated across area under the ROC curve (AUC).

**Results:**

Early recurrence was an independent prognostic risk factor for overall survival (OS) in ICC patients after curative resection (*P*<0.001). The feature importance ranking based on machine learning algorithms showed that AJCC 8th edition N stage, number of tumors, T stage, perineural invasion, and CA125 as the top five variables associated with early recurrence, which was consistent with the independent risk factors of multivariate logistic regression model. Using the aforementioned five variables, we developed four machine learning prediction models, including logistic regression, support vector machine, LightGBM, and random forest. In the training set, the AUC values were 0.849, 0.860, 0.852, and 0.850, respectively. In the testing set, the AUC values were 0.804, 0.807, 0.841, and 0.835, respectively. Among the various prediction models, LightGBM demonstrated superior performance compared to other models in the testing set, exhibiting higher sensitivity, specificity, and accuracy. The effectiveness of ACT on prognosis for different recurrence times, as predicted by the LightGBM model, indicated that ACT could significantly prolong median OS and RFS for ICC patients predicted to experience early recurrence in both the training and testing sets (*P*<0.05). Conversely, for ICC patients predicted to have late recurrence, ACT did not improve OS and RFS (*P*>0.05).

**Conclusion:**

The prediction models established in this study demonstrate good predictive capability and can be used to identify patients who may benefit from ACT.

## Introduction

Intrahepatic cholangiocarcinoma (ICC) accounts for approximately 10% to 12% of all malignant liver tumors (1). Currently, only 20% to 30% of patients are eligible for radical resection, resulting in a poor overall prognosis for patients (2). However, even among patients undergoing radical resection, the 5-year overall survival (OS) rates are also quite low (3, 4). Local or distant recurrence is considered the most significant factor influencing the survival for ICC patients after radical resection, with 42% to 70% of them experiencing recurrence (5). Despite curative resection, tumor recurrence and metastasis are significantly associated with adverse prognostic outcomes. In patients with ICC undergoing radical resection, the 3-year recurrence-free survival (RFS) rate remains below 30%, while the 5-year overall survival rate ranges between 20% and 40% (6–8).

Postoperative recurrence of ICC is associated with multiple factors, including tumor size, lymph node metastasis, microvascular invasion, and R1 resection (9–11). Many studies have confirmed that ICC patients with early recurrence may benefit from adjuvant chemotherapy (ACT) (10, 12, 13). Therefore, identifying high-risk patients for early recurrence and developing individualized treatment strategies will benefit patient survival.

The performance of the machine learning prediction models is more accurate than that of traditional nomogram and TNM staging (13, 14). In recent years, radiomics prediction models have shown improved performance, yet their popularization is constrained by the lack of biological interpretability and practical applicability (15–17). In this study, we developed prediction models to identify early recurrence in ICC patients, while concurrently evaluating the therapeutic potential of ACT for recurrence suppression.

## Methods

### Patients and design

Patients with pathologically proven ICC undergoing curative-intent hepatectomy at the First Affiliated Hospital of Xi’an Jiaotong University between 2013 and 2022 were included. The inclusion criteria were (1): patients underwent curative-intent resection with either microscopically negative or positive margins (R0/R1); (2) detailed records of postoperative recurrence; (3) patients received ACT with comprehensive and systematic regimens; (4) preoperative Child-Pugh grade A or B; and (5) complete clinicopathological data and follow-up information. The exclusion criteria were: (1) patients with hilar cholangiocarcinoma invading the liver; (2) those with mixed cholangiocarcinoma-hepatocellular carcinoma; (3) patients who received neoadjuvant chemotherapy, radiotherapy, chemoradiotherapy, or other treatments for malignant tumors before surgery; and (4) patients who died within 30 days postoperatively. Ultimately, 254 patients were included in the study and staged according to the 8th edition of the AJCC staging system.

### The regimens and indications of ACT

The indications for adjuvant chemotherapy include ICC patients at stages T2 to T4, N1 stage, and those with major vascular invasion, microvascular invasion, or perineural invasion. The selection of chemotherapy regimens primarily adhered to ASCO clinical practice guidelines (18). The chemotherapy regimens comprised four standard 21-day cycles (1): GS: Gemcitabine 1000 mg/m² (days 1,8) + tegafur 40–60 mg bid (days 1-14); (2) GC: Gemcitabine 1000 mg/m² (days 1,8) + cisplatin 30 mg/m² (days 1,8); (3) GEMOX: Gemcitabine 1000 mg/m² (days 1,8) + oxaliplatin 100 mg/m² (day 1); (4) AG: Nab-paclitaxel 125 mg/m² (days 1,8) + gemcitabine 1000 mg/m² (days 1,8).

A total of 71 ICC patients received ACT. 23 (32.4%) patients received gemcitabine + tegafur, 18 (25.4%) patients received gemcitabine + cisplatin, 17 (23.9%) patients received gemcitabine + oxaliplatin,13 (18.3%) patients received nab-paclitaxel+gemcitabine, and postoperative ACT cycles were 5 (2-8) cycles without serious complications during chemotherapy.

### Follow-up

Postoperative surveillance was conducted via outpatient consultations or structured telephone interviews. During the initial 12-month period following resection, patients underwent follow-up assessments at 2- to 3-month intervals, extending to quarterly or semi-annual evaluations thereafter. The surveillance protocol encompassed comprehensive diagnostic modalities including: (1) hepatic function analysis; (2) tumor biomarker quantification (e.g., AFP, CEA, CA19-9); and (3) imaging studies including ultrasound, contrast-enhanced CT, and MRI. Recurrence was defined as the concordant identification of new enhancing lesions on ≥2 independent imaging modalities. Follow-up for all included patients continued through December 2023. Early recurrence occurring within 1 year after surgery is defined as early recurrence (19–21), whereas recurrence occurring after 1 year was considered late recurrence.

### Statistical analysis

Statistical analyses were performed using SPSS (version 25.0) and GraphPad Prism (version 9.0). Continuous variables were expressed as mean ± standard deviation (SD), while categorical variables were compared using Chi-square (*χ*²) tests. Survival outcomes were analyzed through Kaplan-Meier curves with Log-rank tests for univariate comparisons. Variables demonstrating significance (*P*<0.05) in univariate analysis were subsequently entered into a Cox proportional hazards regression model for multivariate analysis. Binary logistic regression was used to identify independent predictors of early recurrence. All statistical tests were two-tailed, and a threshold of *P*<0.05 was deemed statistically significant.

### Development and assessment of machine learning algorithms-based prediction models

The study population was randomized into stratified training (n=178, 70.1%) and testing (n=76, 29.9%) cohorts (**Supplementary Table 1**). Developing prediction models based on the training set (including logistic regression, support vector machine, Light GBM, and random forest), and the testing set was used to evaluate its predictive ability. The importance of the correlation between clinical variables and early recurrence was ranked using the “feature_importance” package in Python software version 3.8. The Bootstrap sampling method and Gini coefficient were used as indicators of feature importance for voting, and the optimal prediction model was obtained through multiple iterations. Evaluating model performance by using the area under the receiver operating characteristic curve (AUC) and confusion matrix.

## Results

The study included 254 patients who had curative-intent resections for histologically confirmed intrahepatic cholangiocarcinoma (ICC) between 2013 and 2022. The overall survival (OS) rates at 1, 3, and 5- years post-resection was 65.7%, 36.0%, and 26.2%, respectively. Corresponding recurrence-free survival (RFS) rates during the same intervals were 47.6%, 25.3%, and 19.2%. The median survival times for the entire cohort were 22.0 months for OS and 12.0 months for RFS.

### Survival analysis comparing early and late recurrence groups

**Figure 1:**
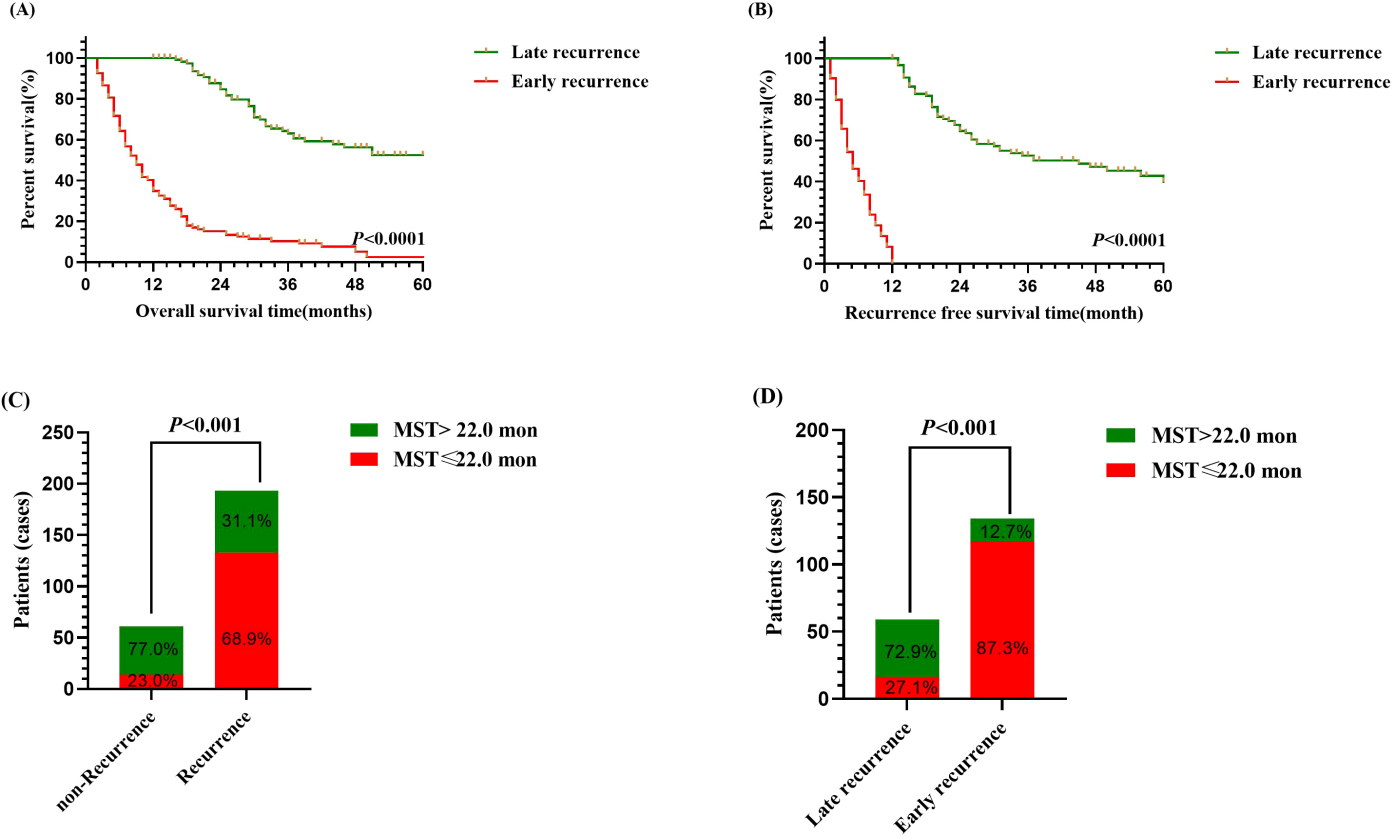
Survival analysis of intrahepatic cholangiocarcinoma patients following curative-intent resection between early and late recurrence groups. **(A)** Kaplan-Meier analysis of OS between early and late recurrence groups. **(B)** Kaplan-Meier analysis of RFS between early and late recurrence groups. **(C)** Bar chart of comparison between non-recurrence and recurrence groups on median OS. **(D)** Bar chart of comparison between early recurrence and late recurrence groups on median OS.

Within the study cohort, 134 patients (52.7%) experienced early recurrence. The early recurrence group showed overall survival (OS) rates of 34.4%, 9.3%, and 2.6% at 1, 3, and 5 years, respectively. In comparison, the late recurrence group demonstrated OS rates of 99.1%, 63.4%, and 49.7% at 1, 3, and 5 years, respectively. The median OS was 9.0 months for the early recurrence group, whereas it was 62.0 months for the late recurrence group (**Figure 1A**, *P*<0.001). Furthermore, the median recurrence-free survival (RFS) was 5.0 months in the early recurrence group, compared to 45.0 months in the late recurrence group (**Figure 1B**, *P*<0.001).

Additionally, patients with recurrence exhibited a significantly higher proportion of median OS ≤ 22.0 months compared to those without recurrence (**Figure 1C**, *P*<0.01), and among them, patients with early recurrence similarly demonstrated a greater proportion of median OS ≤ 22.0 months than patients with late recurrence (**Figure 1D**, *P*<0.01). Thus, the findings indicated that early recurrence negatively impacted the prognosis of patients who underwent curative-intent resection for ICC.

### Prognosis analysis for ICC after curative-intent resection

The survival analysis identified early recurrence as an independent predictor of reduced OS in ICC patients following curative-intent resection compared to late recurrence (*HR*: 7.376, *95% CI*: 4.964–10.960, *P <*0.001).

Additionally, univariate analysis identified ACT as a protective factor for both OS (*HR*: 0.639, *95% CI*: 0.442-0.923, *P*=0.017) and RFS (*HR*: 0.727, *95% CI*: 0.524-0.808, *P*=0.036) in patients with ICC following curative-intent resection. Multivariate analysis further confirmed that ACT was an independent protective factor for OS (*HR*: 0.509, *95% CI*: 0.346-0.749, *P*=0.001) and RFS (*HR*: 0.568, *95% CI*: 0.405-0.797, *P*=0.001). and other details was shown in **Table 1**.

**Table 1:** Univariate and multivariate analysis of prognosis for ICC after curative-intent resection.

	OS				RFS			
	Univariate analysis		Multivariate analysis		Univariate analysis		Multivariate analysis	
	*HR (*95%*CI)*	*P*	*HR (*95%*CI)*	*P*	*HR (*95%*CI)*	*P*	*HR (*95%*CI)*	*P*
Sex								
Female *vs* Male	0.900 (0.661~1.224)	0.501			0.956 (0.719~1.270)	0.754		
Age (year)								
>60 *vs ≤* 60	1.001 (0.736~1.361)	0.997			0.908 (0.684~1.206)	0.504		
Obstructive jaundice								
Yes *vs* No	2.100 (1.368~3.225)	0.001			1.588 (1.041~2.423)	0.032		
HBV infection								
Yes *vs* No	1.030 (0.744~1.425)	0.859			1.043 (0.772~1.408)	0.783		
Hepatolithiasis								
Yes *vs* No	1.765 (1.197~2.604)	0.004			1.330 (0.913~1.937)	0.137		
CEA (ng/ml)								
>5.0 *vs ≤*5.0	1.645 (1.184~2.287)	0.003			1.500 (1.099~2.047)	0.011		
CA19-9(U/ml)								
>39.0 *vs ≤*39.0	1.844 (1.342~2.532)	<0.001	1.563 (1.116~2.188)	0.009	1.405 (1.054~1.872)	0.020		
CA125(U/ml)								
>35.0 *vs ≤*35.0	2.148 (1.565~2.947)	<0.001	1.831 (1.300~2.579)	0.001	1.677 (1.245~2.258)	0.001	1.459 (1.073~1.983)	0.016
Child-Pugh Grade								
Grade B *vs* A	2.356 (1.609~3.450)	<0.001			1.746 (1.202~2.534)	0.003		
Range of liver resection								
Hemi-hepatectomy *vs* Segment resection	1.426 (1.043~1.950)	0.026			1.206 (0.906~1.605)	0.200		
Tumor location								
Right *vs* left	0.832 (0.611~1.132)	0.241			0.915 (0.689~1.215)	0.539		
Number of tumors								
Multiple *vs* Single	1.999 (1.406~2.843)	<0.001			2.220 (1.600~3.080)	<0.001	1.525 (1.076~2.162)	0.018
Tumor differentiation								
Moderate *vs* Well	2.210 (0.966~5.056)	0.060			2.299 (1.120~4.722)	0.023	2.020 (0.966~4.223)	0.062
Poor *vs* Well	2.901 (1.259~6.687)	0.012			2.881 (1.389~5.976)	0.004	2.659 (1.255~5.632)	0.011
Pathological type								
Non-adenocarcinoma *vs* adenocarcinoma	1.686 (0.856~3.320)	0.131			1.134 (0.580~2.219)	0.713		
Tumor size (cm)								
>5.0 *vs ≤* 5.0	1.391 (1.022~1.892)	0.036			1.312 (0.988~1.744)	0.061		
Major vascular invasion								
Yes *vs* No	1.463 (1.022~2.093)	0.038			1.254 (0.892~1.763)	0.192		
Microvascular invasion								
Yes *vs* No	1.475 (0.986~2.207)	0.059			1.426 (0.979~2.077)	0.064		
Perineural invasion								
Yes *vs* No	1.761 (1.202~2.580)	0.004	1.555 (1.117~2.165)	0.009	1.734 (1.220~2.465)	0.002	1.568 (1.084~2.267)	0.017
Surgical margins								
R1 *vs* R0	1.915 (1.179~3.110)	0.009			1.549 (0.982~2.444)	0.060		
AJCC 8th edition T stage								
T_3_ *vs* T_1~2_	1.691 (1.173~2.437)	0.005			1.598 (1.138~2.244)	0.007		
T_4_ *vs* T_1~2_	2.298 (1.558~3.388)	<0.001			1.954 (1.352~2.823)	<0.001		
AJCC 8th edition N stage								
N1 *vs* N0	3.520 (2.515~4.926)	<0.001	1.660 (1.161~2.375)	0.005	3.395 (2.450~4.703)	<0.001	2.205 (1.339~3.630)	0.002
AJCC 8th edition TNM stage								
II *vs* I	1.484 (0.862~2.553)	0.154			1.491 (0.932~2.386)	0.096		
IIIA *vs* I	2.143 (1.336~3.437)	0.002			2.044 (1.335~3.130)	0.001		
IIIB *vs* I	3.985 (2.649~5.994)	<0.001			3.703 (2.556~5.366)	<0.001		
Early recurrence								
Yes vs No	7.947 (5.497~11.491)	<0.001	7.376(4.964~10.960)	<0.001				
Adjuvant chemotherapy								
Yes *vs* No	0.639 (0.442~0.923)	0.017	0.509 (0.346~0.749)	0.001	0.727 (0.524~0.808)	0.036	0.568 (0.405~0.797)	0.001

### Development of machine learning algorithms-based prediction models

**Figure 2:**
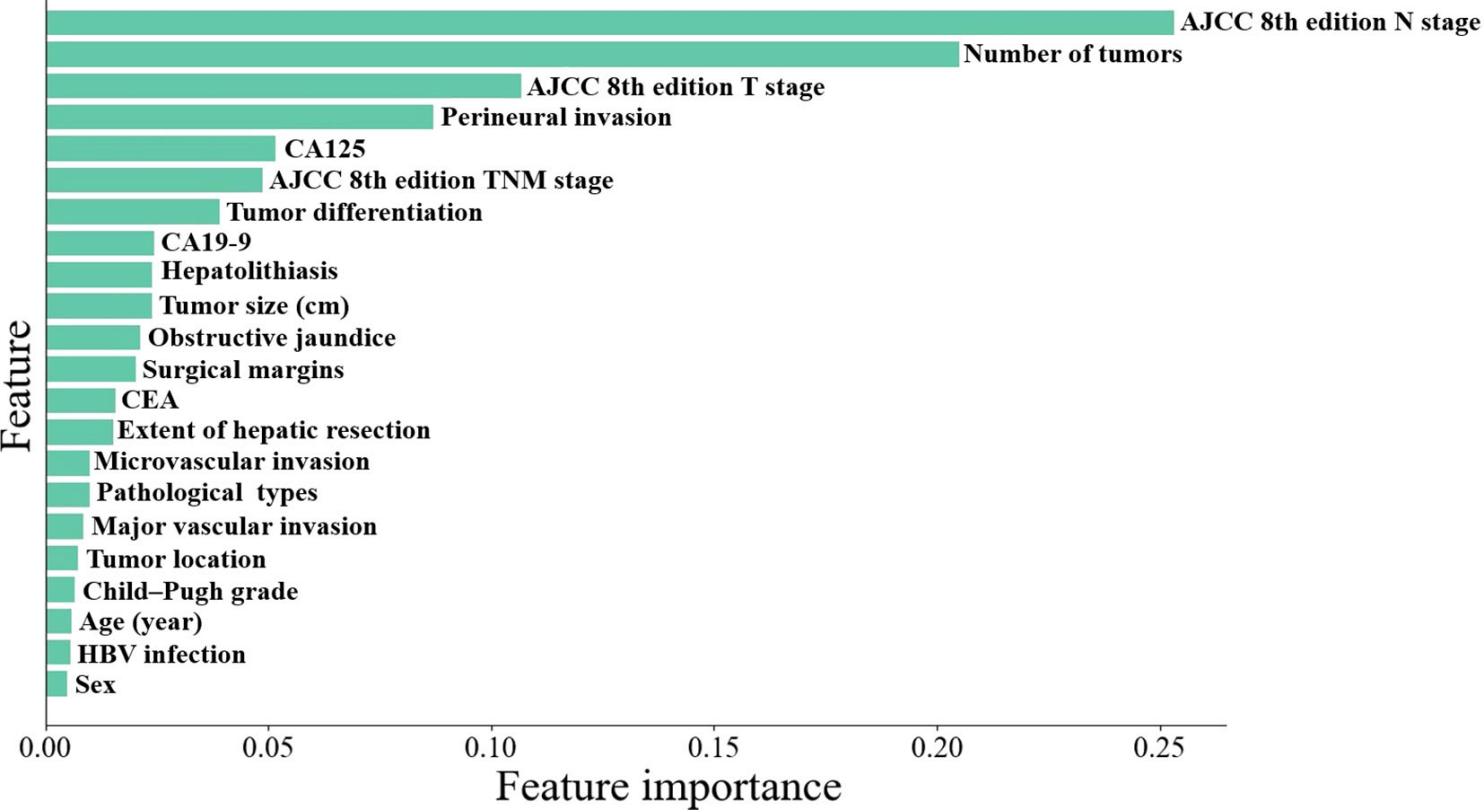
Machine learning algorithms-based importance ranking on variables association to early recurrence.

Univariate and multivariate regression analyses identified CA125 levels, number of tumors, perineural invasion, AJCC 8th edition T stage, and AJCC 8th edition N stage as independent risk factors for early recurrence in ICC patients following curative-intent resection, other details was shown in **Table 2**. Subsequently, the importance ranking based on machine learning algorithms also showed that AJCC 8th edition N stage, number of tumors, AJCC 8th edition T stage, perineural invasion, and CA125 were the top five determinants of early recurrence, revealing concordance with the independent predictors identified through multivariate analysis (**Figure 2**).

**Table 2:** Comparison of clinicopathologic characteristics of early recurrence and late recurrence for ICC after curative-intent resection.

	Late recurrence group	Early recurrence group	Univariate analysis		Multivariate analysis	
	No. (%)	No. (%)	*χ^2^*	*P*	*HR (*95%*CI)*	*P*
Sex						
Male	43 (48.9)	44 (48.9)	0.000	0.997		
Female	45 (51.1)	46 (51.1)				
Age (year)						
≤60	44 (50.0)	44 (48.9)	0.022	0.882		
>60	44 (50.0)	46 (51.1)				
Obstructive jaundice						
No	83 (94.3)	76 (84.4)	4.549	0.033		
Yes	5 (5.7)	14 (15.6)				
HBV infection						
No	56 (63.6)	65 (72.2)	1.507	0.220		
Yes	32 (36.4)	25 (27.8)				
Hepatolithiasis						
No	80 (90.9)	69 (76.7)	6.618	0.010		
Yes	8 (9.1)	21 (23.3)				
CEA (ng/ml)						
≤5.0	70 (79.5)	61 (67.8)	3.171	0.075		
>5.0	18 (20.5)	29 (32.2)				
CA19-9(U/ml)						
≤39.0	49 (55.7)	34 (37.8)	5.731	0.017		
>39.0	39 (44.3)	56 (62.2)				
CA125(U/ml)						
≤35.0	69 (78.4)	56 (62.2)	5.576	0.018	2.992 (1.177~7.602)	0.021
>35.0	19 (21.6)	34 (37.8)				
Child-Pugh grade						
Grade A	80 (90.9)	73 (81.1)	3.538	0.060		
Grade B	8 (9.1)	17 (18.9)				
Range of liver resection						
Segment resection	44 (50.0)	37 (41.1)	1.418	0.234		
Hemi-hepatectomy	44 (50.0)	53 (58.9)				
Tumor location						
Left	41 (46.6)	46 (51.1)	0.364	0.546		
Right	47 (53.4)	44 (48.9)				
Number of tumors						
Single	80 (90.9)	62 (68.9)	13.372	<0.001	4.637 (1.589~13.534)	0.005
Multiple	8 (9.1)	28 (31.1)				
Tumor differentiation						
Well	9 (10.2)	1 (1.1)	9.421	0.009		
Moderate	52 (59.1)	48 (53.3)				
Poor	27 (30.7)	41 (45.6)				
Pathological type						
Adenocarcinoma	85 (96.6)	85 (94.4)	0.478	0.490		
Non-adenocarcinoma	3 (3.4)	5 (5.6)				
Tumor size (cm)						
≤5.0	53 (60.2)	47 (52.2)	1.158	0.282		
>5.0	35 (39.8)	43 (47.8)				
Major vascular invasion						
No	73 (83.0)	72 (80.0)	0.257	0.612		
Yes	15 (17.0)	18 (20.0)				
Microvascular invasion						
No	78 (88.6)	76 (84.4)	0.670	0.413		
Yes	10 (11.4)	14 (15.6)				
Perineural invasion						
No	83 (94.3)	66 (73.3)	14.367	<0.001	3.463 (1.078~11.125)	0.037
Yes	5 (5.7)	24 (26.7)				
Surgical margins						
R0	83 (94.3)	78 (86.7)	3.016	0.082		
R1	5 (5.7)	12 (13.3)				
AJCC 8th edition T stage						
T_1_/T_2_	63 (71.6)	47 (52.2)	9.706	0.008		
T_3_	18 (20.5)	22 (24.4)			1.638 (0.791~3.395)	0.184
T_4_	7 (8.0)	21 (23.3)			4.021 (1.579~10.244)	0.004
AJCC 8th edition N stage						
N0	82 (93.2)	51 (56.7)	31.407	<0.001	7.339 (2.285~23.570)	0.001
N1	6 (6.8)	39 (43.3)				
AJCC 8th edition TNM stage						
I	40 (45.5)	20 (22.2)	41.355	<0.001		
II	20 (22.7)	6 (6.7)				
IIIA	18 (20.5)	14 (15.6)				
IIIB	16 (11.4)	50 (55.6)				

**Figure 3:**
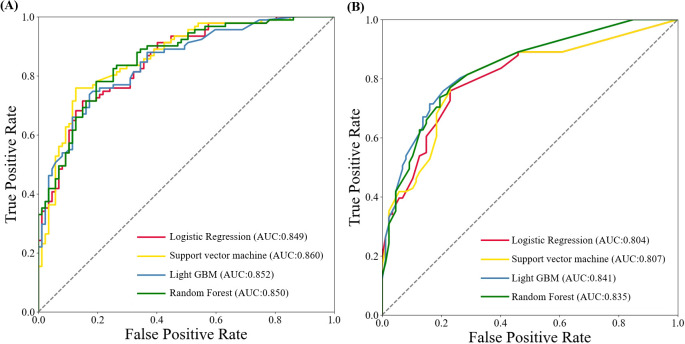
ROC curves of machine learning algorithms-based models for predicting early recurrence of patients with ICC after curative-intent resection. **(A)** ROC curve in training set. **(B)** ROC curve in testing set.

Next, we created four machine learning models: logistic regression, support vector machine, LightGBM, and random forest —using the five identified variables. The AUC values in the training set were 0.849, 0.860, 0.852, and 0.850, respectively. In the testing set, the AUC values were 0.804, 0.807, 0.841, and 0.835, respectively (**Figure 3**). Among above prediction models, Light GBM showed the better performance than other models in the testing set, with higher sensitivity, specificity and accuracy (**Table 3**).

**Table 3:** Confusion matrix of machine learning algorithms-based models for predicting early recurrence of patients with ICC after curative-intent resection.

	Training set			Testing set		
	Sensitivity%	Specificity%	Accuracy%	Sensitivity%	Specificity%	Accuracy%
Logistic regression	79.06	75.00	76.96	76.74	72.82	74.72
Support vector machine	91.86	86.95	89.32	77.78	76.13	76.96
Light GBM	88.50	84.61	86.51	81.25	75.53	78.65
Random forest	82.05	73.00	76.96	77.78	76.13	76.96

### Survival analysis of ICC patients comparing non-ACT and ACT groups based on the predicted classifications

**Figure 4:**
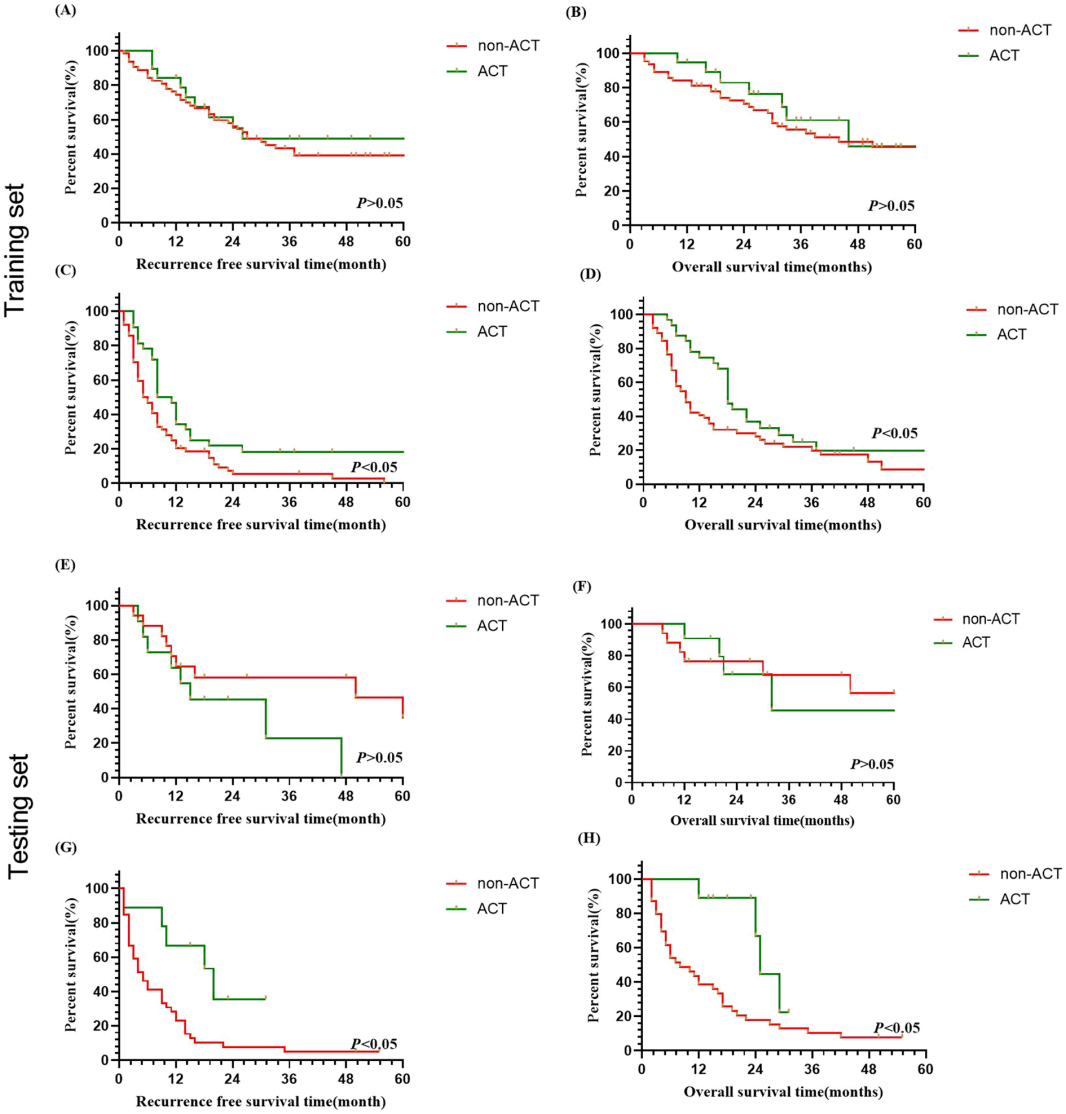
Survival analysis of ICC patients with/without adjuvant chemotherapy in predicted early and late recurrence cohorts after curative resection. **(A, B)** OS and RFS curves for late recurrence in training set. **(C, D)** OS and RFS curves for early recurrence in training set. **(E, F)** OS and RFS curves for late recurrence in testing set. **(G, H)** OS and RFS curves for early recurrence in testing set.

To minimize the impact of varying ACT regimens on patient prognosis, we initially analyzed the differences in outcomes among the four ACT regimens. The results indicated no significant difference in prognosis among the different regimens (*P*> 0.05). Subsequently, we assessed the effectiveness of ACT on the prognosis for different recurrence times based on the predictions of the LightGBM model in ICC patient’s post-curative-intent resection. The results demonstrated that, within the late recurrence group of the training set, the median overall survival (OS) for ICC patients who did not receive ACT was 44.0 months, while it was 46.0 months for those who received ACT, and the median recurrence-free survival (RFS) was 27.0 months compared to 26.0 months, respectively (**Figures 4A, B**, *P*<0.05). In the early recurrence group of the training set, ACT demonstrated significant survival benefit: median OS was prolonged from 9.0 months without ACT to 18.0 months, with parallel improvements in median RFS from 5.5 months to 9.5 months (**Figures 4C, D**, *P*<0.05).

Similarly, the findings also revealed no significant OS or RFS benefit with ACT for ICC patients predicted to have late recurrence. This suggests that ACT may not provide significant benefits for patients with late recurrence (**Figures 4E, F**, *P*>0.05), while ACT could be beneficial to patients who were predicted with early recurrence for OS and RFS (**Figures 4G, H**, *P*<0.05).

## Discussion

Early recurrence emerges as a pivotal prognostic factor influencing outcomes in ICC patients after resection. Meanwhile, accurate identification of early recurrence is essential for tailoring personalized treatment and monitoring strategies for patients. Importantly, Recurrence within one year after surgery may indicate more aggressiveness. A one-year postoperative cut-off is commonly applied to differentiate early from late recurrence. This distinction helps in understanding the underlying tumor characteristics and in tailoring appropriate treatment strategies for ICC patients (10, 19, 20, 22).

Identifying and understanding risk factors for early recurrence can help clinicians stratify patients based on their recurrence risk, tailor follow-up protocols, and implement timely interventions to mitigate recurrence, thereby potentially improving long-term outcomes. Wang et al (10) showed that certain risk factors such as elevated CA 19–9 levels, microvascular invasion, and the presence of multiple tumors were significantly associated with early recurrence of ICC after curative resection. Zhang et al.^12^ identified tumor size, vascular invasion, and lymph node involvement as significant predictors of early recurrence, and they reported that larger tumor sizes and lymph node metastasis significantly correlated with higher early recurrence rates. Our findings corroborate these observations, CA125, number of tumors, perineural invasion, the AJCC 8th edition T stage and N stage were identified as independent risk factors associated with early recurrence., in which CA125, perineural invasion and the AJCC 8th edition N stage were the independent risk factors for the prognosis. Importantly, the aforementioned risk variables were consistent with the top five factors ranked by feature importance in the machine learning algorithms. This robustly underscores their critical roles in predicting early recurrence. Several studies (10, 12, 19, 23–25) have proven that the aforementioned five variables are independent risk factors, providing a solid foundation for establishing a predictive model.

Early recurrence for patients following curative-intent resection can be forcibly predicted by establishing predictive models according to machine learning algorithms. Alaimo et al. (14)constructed prediction models with three machine learning algorithms, with random forest showing the optimal predictive performance in the training and testing sets (AUC: 0.904/0.779). In our study, we constructed four prediction models with similar method, which achieved an average AUC of 0.853 in the training set and 0.822 in the testing set. Notably, Light GBM demonstrated the better performance with an AUC of 0.841 in the testing set. Furthermore, predictive models constructed using radiomics and deep learning based on radiomics, such as CT and MRI have gained significant attention recently, largely due to their superior predictive capabilities. Bo et al. (15) constructed clinical models based on clinicopathological features and CT radiomics models, with mean AUCs of 0.685 and 0.87 ± 0.02, respectively. The CT-based deep learning model for preoperative prediction of early recurrence demonstrated AUCs of 0.998 and 0.994 in the training and validation sets, respectively, without any clinicopathological features being involved in the model construction (26). Therefore, radiomics features have the potential to provide additional information comparable to that available from clinicopathological features to improve the predictive ability. Nevertheless, the limited biological interpretability and the inconvenient use of radiomics models have restricted their popularization in the clinic.

ACT holds promise for reducing early recurrence and improving prognosis in patients with ICC following curative-intent resection. Previous studies have demonstrated that adjuvant chemotherapy (ACT) may improve prognosis (27, 28). Given that multiple factors can influence the efficacy of ACT, identifying patients likely to benefit from it is crucial. Unfortunately, few published studies specifically focus on ACT for patients with resected ICC, due to its low incidence (29). Increasingly, studies are concentrating on the recurrence timing in ICC patients with or without ACT, which can help identify patient groups most likely to benefit from ACT. Many studies (27, 28, 30) have demonstrated that ACT is beneficial for ICC patients following radical resection. However, identifying which patients are most suitable for ACT necessitates further investigation. In this study, the efficacy of ACT on prognosis, based on recurrence time predictions from the LightGBM model, demonstrated that ACT could significantly prolong the median OS and RFS for ICC patients predicted to have early recurrence in both the training and testing sets. Conversely, no statistically significant difference was observed in OS and RFS for patients predicted to have late recurrence after receiving ACT. Thus, accurately predicting early recurrence is crucial for determining suitable treatment strategies for ICC patients’ post-surgery.

However, it is essential to recognize several limitations of the study. Firstly, the retrospective design of our investigation may introduce inherent selection biases, including definition of early recurrence and the differences in ACT treatment cycles due to tolerance or side effects. Secondly, the study cohort was drawn exclusively from tertiary hospital in China, which may limit the broader applicability of our findings to more diverse patient populations. Accordingly, prospective multicenter studies with more diverse populations are needed to validate and refine our prediction models further, and incorporating radiomics and pathogenomics with more comprehensive information could help establish a multimodal prediction model, thereby improving the ability to identify early recurrence. This predictive model can offer more effective decision support for the administration of ACT in ICC patients, helping to tailor treatment strategies based on individual risk profiles and improving overall outcomes.

## Conclusion

In summary, AJCC 8th edition N stage, number of tumors, AJCC 8th edition T stage, perineural invasion, and CA125 as the top five variables associated with early recurrence according to the importance ranking based on machine learning algorithms. The machine learning algorithms utilizing these variables show promising predictive capability and can aid in identifying ICC patients who might benefit from adjuvant chemotherapy after curative-intent resection. We anticipate that our prediction models will assist in identifying appropriate patients who could benefit from ACT, thereby prolonging survival time for ICC patients following curative-intent resection.

## Data Availability

The original contributions presented in the study are included in the article/**Supplementary Material**. Further inquiries can be directed to the corresponding author.
