# Supplementary material for: Who benefits from adjuvant chemotherapy? Identification of early recurrence in intrahepatic cholangiocarcinoma patients after curative-intent resection using machine learning algorithms
Source: Front Oncol. 2025 Jun 6;15:1594200. doi: 10.3389/fonc.2025.1594200 (PMC12178869; doi:10.3389/fonc.2025.1594200)
Supplement: Supplementary file 1 [file Table1.docx]

**Supplemental table 1** Demographics and clinical characteristics of ICC patients between training and testing sets

|  | Case | Training set | Testing set | *χ^2^* | | *P* |
| --- | --- | --- | --- | --- | --- | --- |
| Sex |  |  |  |  | |  |
| Male | 132 (52.0) | 87 (48.9) | 45 (59.2) | 2.279 | | 0.131 |
| Female | 122 (48.0) | 91 (51.1) | 31 (40.8) |  |  |  |
| Age (year) |  |  |  |  | |  |
| ≤55 | 131 (51.6) | 88 (49.4) | 43 (56.6) | 1.097 | | 0.297 |
| >55 | 123 (48.4) | 90 (50.6) | 33 (43.4) |  |  |  |
| Obstructive jaundice |  |  |  |  | |  |
| No | 226 (89.0) | 159 (89.3) | 67 (88.2) | 0.074 | | 0.785 |
| Yes | 28 (11.0) | 19 (10.7) | 9 (11.8) |  |  |  |
| HBV infection |  |  |  |  | |  |
| No | 172 (67.7) | 121 (68.0) | 51 (67.1) | 0.019 | | 0.892 |
| Yes | 82 (32.3) | 57 (32.0) | 25 (32.9) |  |  |  |
| Hepatolithiasis |  |  |  |  | |  |
| No | 215 (84.6) | 149 (83.7) | 66 (86.8) | 0.403 | | 0.526 |
| Yes | 39 (15.4) | 29 (16.3) | 10 (13.2) |  |  |  |
| CEA (ng/ml) |  |  |  |  | |  |
| ≤5.0 | 186 (73.2) | 131 (73.6) | 55 (72.4) | 0.041 | | 0.840 |
| >5.0 | 68 (26.8) | 47 (26.4) | 21 (27.6) |  |  |  |
| CA19-9(U/ml) |  |  |  |  | |  |
| ≤39.0 | 118 (46.5) | 83 (46.6) | 35 (46.1) | 0.007 | | 0.923 |
| >39.0 | 136 (53.5) | 95 (53.4) | 41 (53.9) |  |  |  |
| CA125(U/ml) |  |  |  |  | |  |
| ≤35.0 | 172 (67.7) | 125 (70.2) | 47 (61.8) | 1.712 | | 0.191 |
| >35.0 | 82 (32.3) | 53 (29.8) | 29 (38.2) |  |  |  |
| Child-Pugh Grade |  |  |  |  | |  |
| Grade A | 218 (85.8) | 153 (86.0) | 65 (85.5) | 0.008 | | 0.929 |
| Grade B | 36 (14.2) | 25 (14.0) | 11 (14.5) |  |  |  |
| Range of liver resection |  |  |  |  | |  |
| Segment resection | 118 (46.5) | 81 (45.5) | 37 (48.7) | 0.216 | | 0.642 |
| Hemi-hepatectomy | 136 (53.5) | 97 (54.5) | 39 (51.3) |  |  |  |
| Tumor location |  |  |  |  | |  |
| Left | 125 (49.2) | 87 (48.9) | 38 (50.0) | 0.027 | | 0.870 |
| Right | 129 (50.8) | 91 (51.1) | 38 (50.0) |  |  |  |
| Number of tumors |  |  |  |  | |  |
| Single | 194 (76.4) | 142 (79.8) | 52 (68.4) | 3.806 | | 0.051 |
| Multiple | 60 (23.6) | 36 (20.2) | 24 (31.6) |  |  |  |
| Tumor differentiation |  |  |  |  | |  |
| Well | 16 (6.3) | 10 (5.6) | 6 (7.9) | 0.542 | | 0.763 |
| Moderate | 143 (56.3) | 100 (56.2) | 43 (56.6) |  |  |  |
| Poor | 95 (37.4) | 68 (38.2) | 27 (35.5) |  |  |  |
| Pathological type |  |  |  |  | |  |
| Adenocarcinoma | 241 (94.9) | 170 (95.5) | 71 (93.4) | 0.477 | | 0.490 |
| Non-adenocarcinoma | 13 (5.1) | 8 (4.5) | 5 (6.6) |  |  |  |
| Tumor size (cm) |  |  |  |  | |  |
| ≤5.0 | 143 (56.3) | 100 (56.2) | 43 (56.6) | 0.003 | | 0.953 |
| >5.0 | 111 (43.7) | 78 (43.8) | 33 (43.4) |  |  |  |
| Major vascular invasion |  |  |  |  | |  |
| No | 199 (78.3) | 145 (81.5) | 54 (71.1) | 3.401 | | 0.015 |
| Yes | 55 (21.7) | 33 (18.5) | 22 (28.9) |  |  |  |
| Microvascular invasion |  |  |  |  | |  |
| No | 214 (84.3) | 154 (86.5) | 60 (78.9) | 2.300 | | 0.129 |
| Yes | 40 (15.7) | 24 (13.5) | 16 (21.1) |  |  |  |
| Perineural invasion |  |  |  |  | |  |
| No | 208 (81.9) | 149 (83.7) | 59 (77.6) | 1.326 | | 0.250 |
| Yes | 46 (18.1) | 29 (16.3) | 17 (22.4) |  |  |  |
| Surgical margins |  |  |  |  | |  |
| R0 | 229 (90.2) | 161 (90.4) | 68 (89.5) | 0.057 | | 0.811 |
| R1 | 25 (9.8) | 17 (9.6) | 6 (10.5) |  |  |  |
| AJCC 8th edition T stage |  |  |  |  | |  |
| T_1_/T_2_ | 153 (60.2) | 110 (61.8) | 43 (56.6) | 2.285 | | 0.319 |
| T_3_ | 55 (21.7) | 40 (22.5) | 15 (19.7) |  |  |  |
| T_4_ | 46 (18.1) | 28 (15.7) | 18 (23.7) |  |  |  |
| AJCC 8th edition N stage |  |  |  |  | |  |
| N0 | 192 (75.6) | 133 (74.7) | 59 (77.6) | 0.245 | | 0.621 |
| N1 | 62 (24.4) | 45 (25.3) | 17 (22.4) |  |  |  |
| AJCC 8th edition TNM stage |  |  |  |  | |  |
| I | 83 (32.7) | 60 (33.7) | 23 (30.3) | 1.689 | | 0.639 |
| II | 42 (16.5) | 26 (14.6) | 16 (21.1) |  |  |  |
| IIIA | 44 (17.3) | 32 (18.0) | 12 (15.8) |  |  |  |
| IIIB | 85 (33.5) | 60 (33.7) | 25 (32.9) |  |  |  |
| Early recurrence |  |  |  |  | |  |
| No | 121 (47.6) | 88 (49.4) | 33 (43.4) | 0.773 | 0.379 | |
| Yes | 133 (52.4) | 90 (50.6) | 43 (56.6) |  |  |  |
| Adjuvant chemotherapy |  |  |  |  | |  |
| No | 183 (72.0) | 126 (70.8) | 57 (75.0) | 0.470 | | 0.493 |
| Yes | 71 (28.0) | 52 (29.2) | 19 (25.0) |  |  |  |
